# Systemic inflammatory response after robotic versus laparoscopic abdominal surgery: a systematic review and meta-analysis with colorectal cancer subgroup analysis

**DOI:** 10.1007/s11701-026-03527-x

**Published:** 2026-06-01

**Authors:** Taya Keating, C. Drumm, Niall Kennedy, C. Cullinane, E. Condon, M. Fitzgerald, C. Peirce, J. Calvin Coffey, Christina A. Fleming

**Affiliations:** 1 Royal College of Surgeons Ireland (RCSI), 123 St Stephens Green, Dublin 2, Ireland; 2 Department of Colorectal Surgery, University Hospital Limerick, Dooradoyle, Limerick Ireland; 3 Department of Anaesthesia, University Hospital Limerick, Dooradoyle, Limerick Ireland; 4 School of Medicine, University of Limerick, Limerick, Ireland

**Keywords:** Robotic surgery, Laparoscopic surgery, Colorectal surgery, Systemic inflammatory response, c-reactive protein

## Abstract

Robotic surgery offers enhanced precision that can reduce tissue trauma and the systemic inflammatory response compared with laparoscopy. Clinically, a lower inflammatory response is associated with reduced postoperative pain, fewer complications, shorter hospital stay and improved oncological outcomes. This systematic review and meta-analysis compared postoperative trends in biochemical markers of the systemic inflammatory response in laparoscopic versus robotic colorectal surgery. This review was prospectively registered on PROSPERO (CRD420251167614) and reported in accordance with PRISMA guidelines. MEDLINE (PubMed), EMBASE (OvidSP), and Cochrane CENTRAL were searched for primary studies reporting biochemical surrogates of the systemic inflammatory response in adult patients following elective abdominal surgery. Eligible studies were then compared and meta-analysis performed using RevMan(Cochrane, Version 7.2.0). Eight studies (two randomised controlled trials and six observational cohort studies with an overall moderate risk of bias) involving 1,706 patients (*n* = 714 robotic, *n* = 992 laparoscopic) met inclusion criteria. C-reactive protein (CRP), albumin, and white cell count (WCC) were the most frequently reported markers of inflammation reported. Postoperative day 3 CRP was significantly lower after robotic surgery compared with laparoscopy [MD − 13.18 mg/l (95% CI − 22.29, − 4.08), *p* = 0.005), this was further demonstrated in colorectal cancer surgery subgroup analysis [MD -17.22 (95%CI -26.28, -8.17), *p* = 0.0002 I²= 0%]. Among secondary outcome measures, robotic surgery was associated with a significantly lower complication rate (*p* = 0.02) and a non-significant trend toward reduced intraoperative blood loss (*p* = 0.15), despite longer operative duration (mean difference 24 min). There was a non-significant trend towards shorter length of hospital stay with robotic surgery (*p* = 0.16). This meta-analysis demonstrated that post-operative CRP, a biochemical marker of peri-operative inflammation, was significantly lower in robotic surgery compared with laparoscopy. These findings support the hypothesis that robotic surgery potentially minimises tissue trauma and may contribute to reduced peri-operative inflammation.

**What does this add to the literature?**

While emerging evidence suggests that robotic colorectal surgery may elicit a less pronounced postoperative inflammatory response than conventional laparoscopic surgery, prior to this study there lacked a meta-analysis combining all available post robotic colorectal surgery post-operative inflammatory response data. The enhanced dexterity, tremor filtration, and improved three-dimensional visualisation offered by the robotic platform may facilitate more precise tissue handling and minimise collateral tissue trauma, however the precise biological mechanism behind this is not yet fully understood. Consequently, robotic surgery has been associated with lower postoperative inflammatory markers and a reduced physiological stress response in some studies. The implications of a lower systemic inflammatory response post robotic surgery could potentially influence choice of approach for specifically colorectal cancer patients, where a lower systemic inflammatory response, independent of complete resection (R0) and complication rates is associated with better oncological outcomes and survival.

**Supplementary Information:**

The online version contains supplementary material available at 10.1007/s11701-026-03527-x.

## Introduction

The adoption of robotic surgery has transformed colorectal surgery over the past decade. Robotic surgical platforms offer superior dexterity and range of motion with seven degrees of freedom and 540° range of motion in comparison with the straight instruments of traditional laparoscopy. This allows for improved access in confined spaces such as the pelvis [1, 2]. Once the robotic arms are targeted, the ports are fixed in place and act as a fulcrum thus there is less musculoskeletal torque as a result. The robotic surgery platforms filter out hand tremors and offers motion scaling, allowing for enhanced fine motor control and precision during dissection. The high-definition 3D camera offers superior visualisation and depth perception, in comparison with the conventional 2D laparoscopic camera. In addition, robotic platforms stabilise the camera and the view is controlled by the operating surgeon, versus the potential for drift or suboptimal view by a novice assistant. It is plausible that these technological advantages may collectively improve operative dexterity and are hypothesised to reduce tissue stress and overall systemic inflammatory stress response to surgery, however the precise biological mechanism behind this is not yet fully understood. Consequently, robotic surgery has been associated with lower postoperative inflammatory markers and a reduced physiological stress response in some studies [3, 4]. The potential implications of a lower systemic inflammatory response post robotic surgery could influence choice of approach for specifically colorectal cancer patients, where a lower systemic inflammatory response, independent of an R0 resection and complication rates could potentially positively impact on oncological and survival outcomes, however this oncological relationship with the inflammatory response is not directly examined in this meta-analysis [5–8].

Trends towards a reduction in intra-operative blood loss, complication rates and length of stay have been reported following robotic surgery in comparison with laparoscopy [9–22]. The enhanced precision afforded by the robotic console combined with superior visualisation is suggested to reduce tissue trauma thus minimising local tissue stress and inflammation [23]. These advantages translate into meaningful clinical benefits for patients, particularly less postoperative pain, shorter hospitalisation, lower conversion-to-open rates, and fewer complications compared with the laparoscopic approach [24–29]. With widespread adoption of robotic surgery, studies are now assessing the underlying physiological stress response to robotic surgery compared to laparoscopy, and the hypothesis that a reduced systemic inflammatory response is observed following robotic surgery, demonstrated by evaluating markers of the systemic inflammatory response such as post-operative White Cell Count (WCC), C-Reactive Protein (CRP), albumin (inverse surrogate of inflammation) and cytokines such as interleukins IL-6 and IL-10 [10–16, 30, 31].

In the context of these emerging reports, to date there is a lack of a systematic review or meta-analysis synthesising the available evidence on the systemic inflammatory response post robotic surgery compared to laparoscopic surgery. This paper aims to address this gap in the literature, hypothesising that robotic-assisted abdominal surgery is associated with a lower systemic inflammatory response in comparison with laparoscopy. The enhanced dexterity, tremor filtration, and improved three-dimensional visualisation offered by the robotic platform may facilitate more precise tissue handling and minimise collateral tissue trauma, however the precise biological mechanism behind this is not yet fully understood. By using surrogates of inflammation such as CRP, WCC or albumin levels (inverse surrogate) following robotic and laparoscopic surgery, the relationship with perioperative inflammation following robotic and laparoscopic colorectal surgery is examined in this study. Given the demonstrated negative impact of an exaggerated systematic inflammatory response peri-operatively has on cancer outcomes, a subgroup analysis in colorectal cancer surgery was also performed.

## Methods

This systematic review was conducted in accordance with PRISMA guidelines (2020) and prospective registration was performed on PROSPERO (CRD420251167614) on 13th October 2025. The review was completed on 14th December 2025.

### Search strategy

An electronic search was conducted using MEDLINE (PubMed), EMBASE (OvidSP) and Cochrane CENTRAL databases. All studies published in the last ten years, between 2015 and 2025 were included in the search, as this time period would capture the most up to date current studies since the widespread adoption of the robotic surgery platform. Conference abstracts, grey literature, preprints, trial registries and non-English studies were not included. MeSH terms used were as follows: ((robotic assisted surgery) AND (laparoscopic) AND (c reactive protein)) / ((robotic assisted surgery) AND (laparoscopic) AND (inflammation)) / ((robotic assisted surgery) AND (laparoscopic) AND (systemic inflammatory response syndrome)). The references of each included study were also reviewed to ensure all relevant studies were included. All titles were initially screened and appropriate abstracts were reviewed by two independent reviewers (TK and NK). A third reviewer (CF) was nominated to resolve any disagreements. The date of last search was 13th October 2025.

### Risk of bias

Risk of bias assessment was performed using Cochrane Risk of Bias 2 (RoB2) [32] for randomized controlled trials and Risk of Bias in Non-Randomized Studies - of Interventions (ROBINS-1) [33] for retrospective cohort studies. In addition, the Risk of Bias due to Missing Evidence (ROBME) tool was used to assess for risk of bias due to missing results [34]. The quality of evidence and strength of recommendations was analysed based on the Grading of Recommendations Assessment, Development and Evaluation (GRADE guidelines 26) system [35].

### Inclusion criteria

All studies (randomised control trials (RCTs), observational cohort studies, case series) meeting the following inclusion criteria were analysed: adult patients undergoing elective colorectal/abdominal surgery; both benign and cancer surgery, in which any biochemical measure of post-operative inflammation, comparing laparoscopic versus robotic surgery was reported.

### Exclusion criteria

Paediatric patients or those who underwent open or emergency surgery were excluded, as were non-general surgery patients. Studies that did not present raw data results for CRP or WCC or albumin (e.g., studies that used inflammatory indices such as systemic immune inflammation index (SII)) were excluded as combining data for meta-analysis would not be possible.

### Data extraction

All titles were initially screened for eligibility and appropriate abstracts were reviewed by two independent reviewers (TK and NK). A third reviewer (CF) was nominated to resolve any disagreements by way of discussion with the two reviewers. The references of each included study were also screened to ensure all relevant studies were identified for inclusion in the meta-analysis.

### Outcomes

The primary outcome was comparison of biochemical surrogates of post-operative inflammatory response in laparoscopic compared to robotic surgery. This was defined as post operative CRP. The secondary outcome measures analysed were post operative serum WCC and albumin levels as well as complication rates, length of stay, intra-operative blood loss and operative duration. Major complications (Clavien–Dindo (CD) > II morbidities) was additionally analysed as these complications reflect the need for further intervention, which impacts the post-operative inflammatory response and duration of hospital stay. A subgroup analysis was performed on colorectal cancer patients.

### Statistical analysis

Meta-analysis was performed using RevMan (Cochrane, Version 7.2.0) [36]. Random-effects meta-analysis was performed using the inverse-variance method with restricted maximum likelihood estimation of between-study variance. Continuous outcomes (e.g. CRP, length of stay, blood loss, operative duration) were reported as mean with standard deviations. In papers that did not publish this data (e.g., in studies reporting median and interquartile ranges), the reviewers contacted corresponding authors by email for the raw data values to ensure data completeness. Mean differences (MD) with 95% confidence intervals (CI) were calculated using RevMan (Cochrane, Version 7.2.0) [36]. Dichotomous outcomes (e.g., complication rates) were reported using risk ratios (RR) with 95% confidence intervals. Heterogeneity, using a random-effects model was assessed using I² statistics, with > 25% being considered significant heterogeneity. P values of < 0.05 were considered significant.

## Results

### Study characteristics

**Fig. 1:**
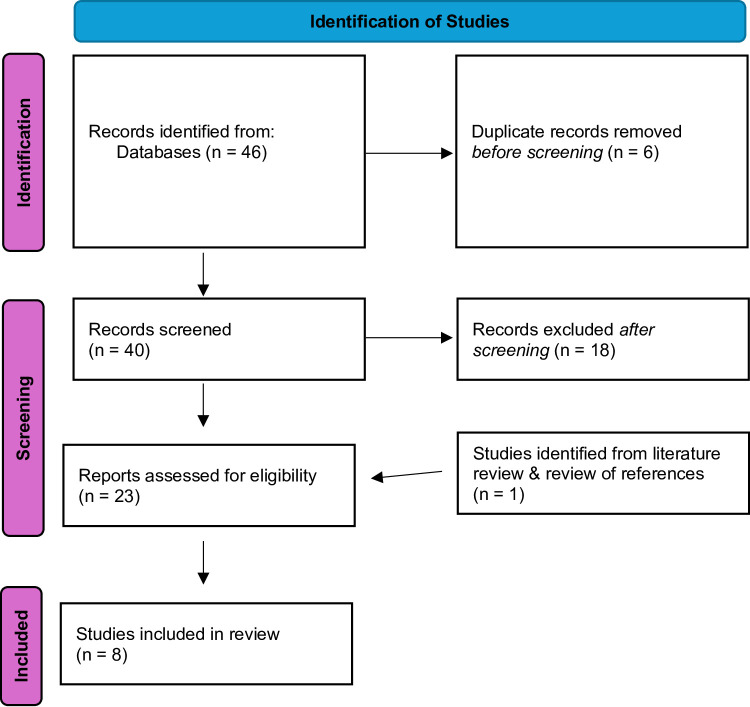
Study selection

The search as outlined above yielded forty-six studies. Duplicate studies (*n* = 6) and urology/gynaecology/paediatric studies (*n* = 18) were removed and the remaining twenty-two studies were screened (Fig. 1). Seven studies were identified to meet the inclusion criteria and were included in the systematic review [10–13, 15, 16, 31]. During the review of references, one further study was identified as suitable for inclusion [14], bringing the total to eight studies for inclusion in this systematic review and meta-analysis (Table 1).

**Table 1:** Summary of characteristics of studies selected for inclusion

Authors	Year	Name of study	Type of study	Numbers of Participants	Primary Outcome: SIRS response
Čuk P, et al.	2024	Systemic inflammatory response in robot-assisted and laparoscopic colorectal surgery (SIRIRALS)	Randomised control trial	50 patients 25 laparoscopic & 25 robotic-assisted colorectal cancer resections	Lower CRP D1 post robotic surgery
Valorenzos A, et al.	2025	Inflammatory response and short-term outcomes after laparoscopic versus robotic transabdominal preperitoneal inguinal hernia repair: randomized clinical trial (ROLAIS).	Randomised control trial	139 patients 65 laparoscopic hernia repairs (TAPP) & 74 robotic-assisted hernia repairs (TAPP)	Lower CRP D1 and D3 post robotic surgery IL-6 lower post robotic surgery
Ingham AR, Campbell Roxburgh et al.	2024	Robotic-assisted surgery for left-sided colon and rectal resections is associated with reduction in the postoperative surgical stress response and improved short-term outcomes: a cohort study	Retrospective cohort study	1031 patients 376 laparoscopic & 172 robotic-assisted colorectal cancer patients **additional 483 open surgery patients in the study	Lower CRP day 1–4 post robotic surgery
Ishiyama Y, et al.	2025	Comparative analysis of postoperative inflammation and pain: robot-assisted versus single-incision laparoscopic surgery for right-sided colon cancer	Retrospective cohort study	180 patients 67 laparoscopic & 67 robotic-assisted right sided colon cancer patients (after propensity matching)	Lower CRP/albumin ratio post robotic surgery
Widder A, et al.	2022	Robotic-Assisted versus Laparoscopic Left Hemicolectomy-Postoperative Inflammation Status, Short-Term Outcome and Cost Effectiveness.	Retrospective cohort study	61 patients 35 laparoscopic & 26 robotic-assisted left hemicolectomies for both cancer & benign indications	Lower CRP day 3 and 5 post robotic surgery
Park EJ, et al.	2024	Robotic surgery may lead to reduced postoperative inflammatory stress in colon cancer: a propensity score–matched analysis	Retrospective cohort study	132 patients 66 laparoscopic & 66 robotic-assisted colorectal cancer (primary surgery)	Equivocal WCC/neut post robotic surgery Better PNI post robotic surgery
Kim HS, et al.	2023	Short-term outcomes of single-incision robotic colectomy versus conventional multiport laparoscopic colectomy for colon cancer	Retrospective cohort study	140 patients 97 laparoscopic & 43 robotic-assisted colorectal cancer (primary surgery)	Lower maximal CRP (measured on days 1–5
Seo WJ, et al.	2020	Reduced-port totally robotic distal subtotal gastrectomy for gastric cancer: 100 consecutive cases in comparison with conventional robotic and laparoscopic distal subtotal gastrectomy	Retrospective cohort study	602 patients 261 laparoscopic & 241 robotic-assisted gastrectomies for gastric cancer **For the purpose of this meta-analysis we analysed the conventional approach rather than the 100 patients in the reduced port robotic-assisted group	Equivocal post-operative CRP or WCC (conventional robotic versus conventional laparoscopic approach)

Two studies were randomised control trials, six were retrospective observational cohort studies, all published between 2020 and 2025 (Table 1). This amounted to a total of 1,706 patients, subdivided into 714 (42%) robotic cases and 992 (58%) laparoscopic cases, with an overall gender balance of 61% (*n* = 1045) male and 39% (*n* = 661) female. For CRP data analysis, six studies (reporting on 1,435 patients) had sufficient data to be compared, including studies where the authors provided mean and standard deviation via email (where median or inflammation indices or ratios were reported in published papers). Sub-analysis of colorectal cancer patients was performed including five studies, reporting on 1,004 patients [11, 12, 14, 15, 31]. For the colorectal cancer subgroup analysis of CRP, four studies (*n* = 872 patients) had sufficient data for analysis.

### Risk of bias and certainty of evidence

Overall risk of bias across all eight studies was defined as moderate using study design-specific assessment tools as described with a full summary and interpretation in the Supplementary Data file.

Potential areas for bias within the SIRIRALS study included selection bias, a risk of missing outcome data and in addition IL-6 (pre-defined primary outcome measure) and length of stay (pre-defined secondary outcome measure) were not included in the analysis thus leaving potential for selective reporting.

The open-label nature of the ROLAIS trial allows for some performance bias surrounding peri-operative decision making. Furthermore, the study began in November 2022, however, was retrospectively registered on ClinicalTrials.gov in February 2023. The six cohort studies have a moderate quality of evidence and strength of recommendation, largely due to their single centre observational nature thus allowing for some confounding bias and additional potential for selective reporting due to their retrospective nature.

With regard to risk of bias due to missing results; only one of the cohort studies states that follow up was complete for all participants. Both randomised control trials state no participants were lost to follow up. For the remaining five cohort studies there is a risk of bias due to missing participant and outcome data in this regard. The majority of the studies included in this systematic review were of moderate to large sample size thus reducing bias and overestimation of treatment effects.

#### Primary outcome measures: post-operative inflammatory response (Fig. 2)

**Fig. 2:**
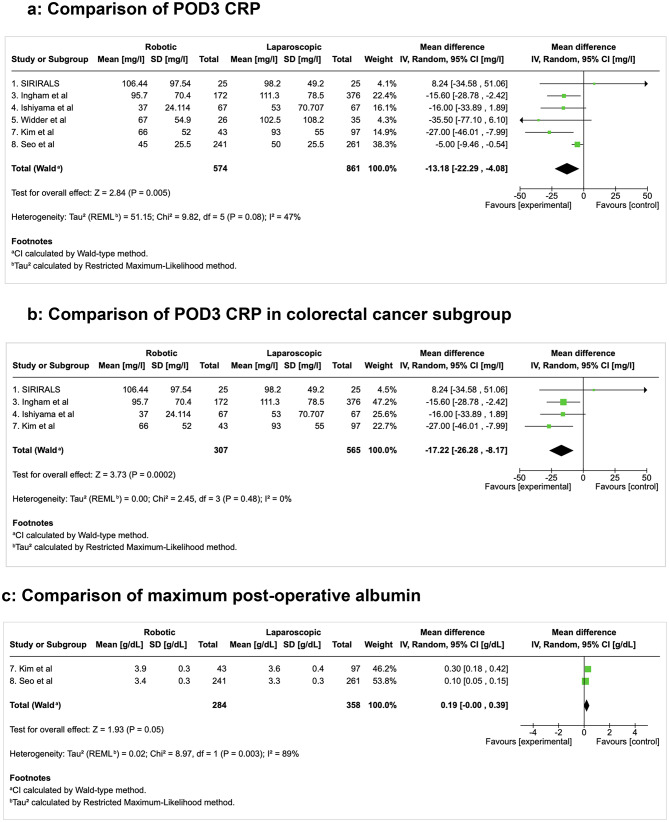
Comparison of post-operative CRP and Albumin. CRP; C-reactive protein, POD; post-operative day

In pooled meta-analysis, there were six studies that included sufficient data for comparison of post-operative CRP levels (*n* = 1,435 patients) as a surrogate for post-operative inflammation [11–13, 15, 16, 31]. Robotic surgery patients had significantly lower CRP on post-operative day three (POD3) than patients undergoing laparoscopy with a mean difference (MD − 13.18 mg/l, 95%CI [–22.29, − 4.08], *p* = 0.005). The heterogeneity was moderate (I² = 47%) and may be due to the procedural heterogeneity (both benign and cancer operations). It is for this reason that a specific sub-analysis on colorectal cancer operations alone was conducted (see below). While there was a trend towards lower CRP levels on POD1, this did not reach significance (MD -6.27, 95% CI [-13.17, 0.62], *p* = 0.07).

Maximum serum albumin levels (as an inverse surrogate for post-operative inflammation) were higher in the robotic surgery group, albeit only reaching borderline significance, in the two studies (*n* = 642 patients) that reported albumin post-operatively (MD 0.19, 95% CI [-0.00, 0.39], *p* = 0.05, I² = 89%).

WCC did not significantly vary between the two approaches in the four studies (*n* = 835 patients) that included WCC measurements (MD -0.04, 95% CI [-0.50, 0.42], *p* = 0.87, I² = 0%).

#### Subgroup analysis on colorectal cancer patients (Fig. 2a-c)

Four studies included sufficient CRP data for comparison of post-operative CRP in colorectal cancer surgery specifically (*n* = 872 patients; *n* = 307 (35%) robotic cases, *n* = 565 (65%) laparoscopic cases) [11, 12, 15, 31]. Within this subgroup, pooled meta-analysis demonstrated that POD3 CRP was significantly lower in the robotic group compared to the laparoscopic group with no heterogeneity demonstrated between the groups (MD -17.22, 95%CI [-26.28, -8.17], *p* = 0.0002, I² = 0%). While CRP levels on day one post op tended to be lower in the colorectal cancer studies also, this did not reach significance in pooled meta-analysis (MD -7.93, 95% CI [-20.73, 4.86], *p* = 0.22, I² = 84%). The was high heterogeneity, potentially due to procedural heterogeneity, differences in surgical technique and experience, patient and disease heterogeneity and variations in peri-operative care.

#### Secondary outcome measures: operation duration (Fig. 3a-b)

**Fig. 3:**
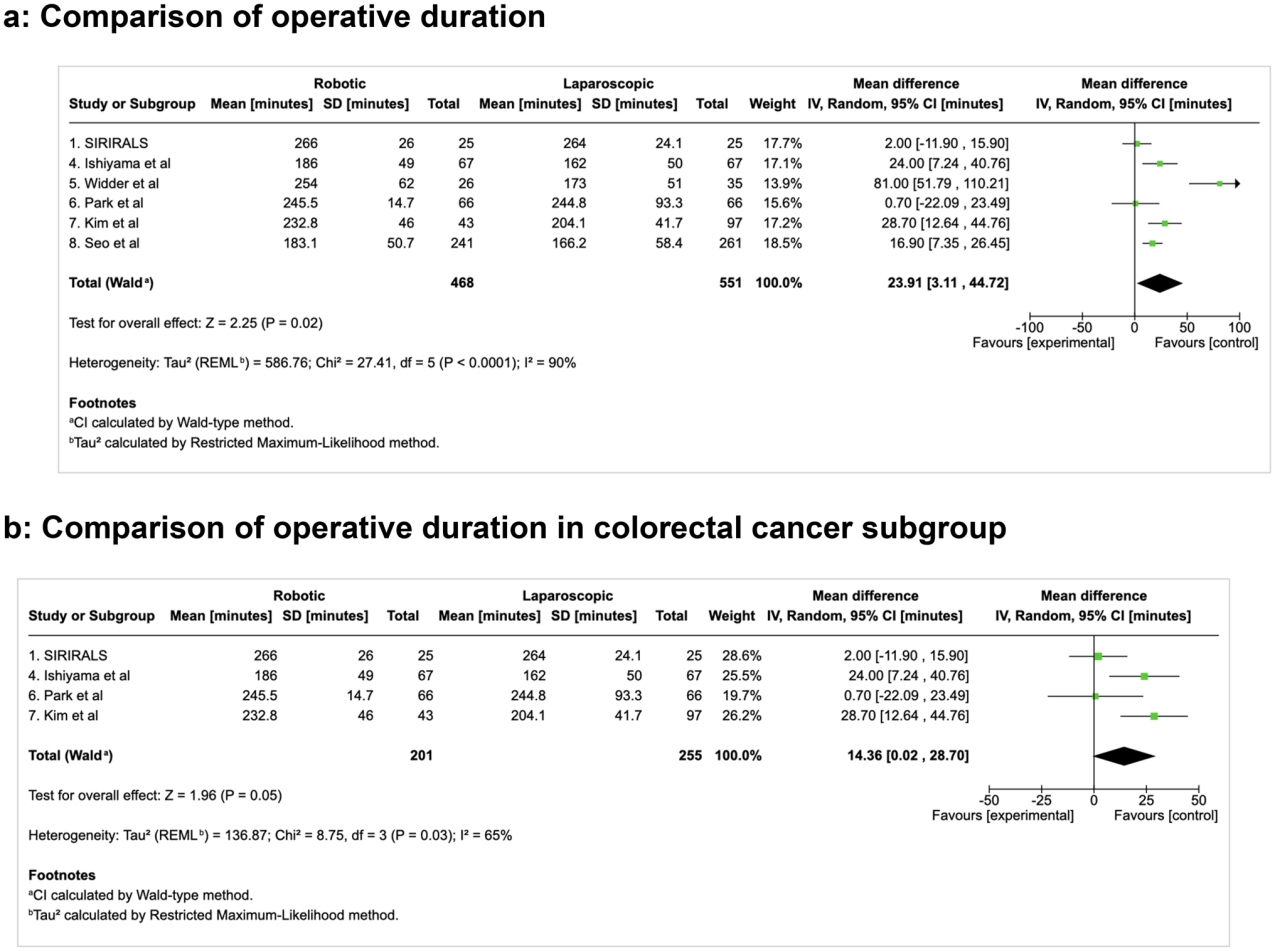
Comparison of operative duration

In keeping with previous published studies, the pooled analysis of 1,019 patients showed a statistically significantly longer operative time with the robotic approach, with a mean difference of 24 min and significant heterogeneity noted (MD 23.91, 95%CI [3.11, 44.72], *p* = 0.02; I² = 90%). The heterogeneity was significant (I² = 90%) and may be due to the procedural heterogeneity (both benign and cancer operations) included in the meta-analysis, as well as differences in surgical technique and experience, patient and disease heterogeneity and variations in peri-operative care.

This difference in operative duration was less pronounced in the subgroup analysis of colorectal cancer patients (*n* = 456), with a mean difference of 14 min; only reaching borderline significance (MD 14.36, 95%CI [0.02, 28.70], *p* = 0.05; I² = 65%).

#### Secondary outcome measures: complication rates (Fig. 4a)

**Fig. 4:**
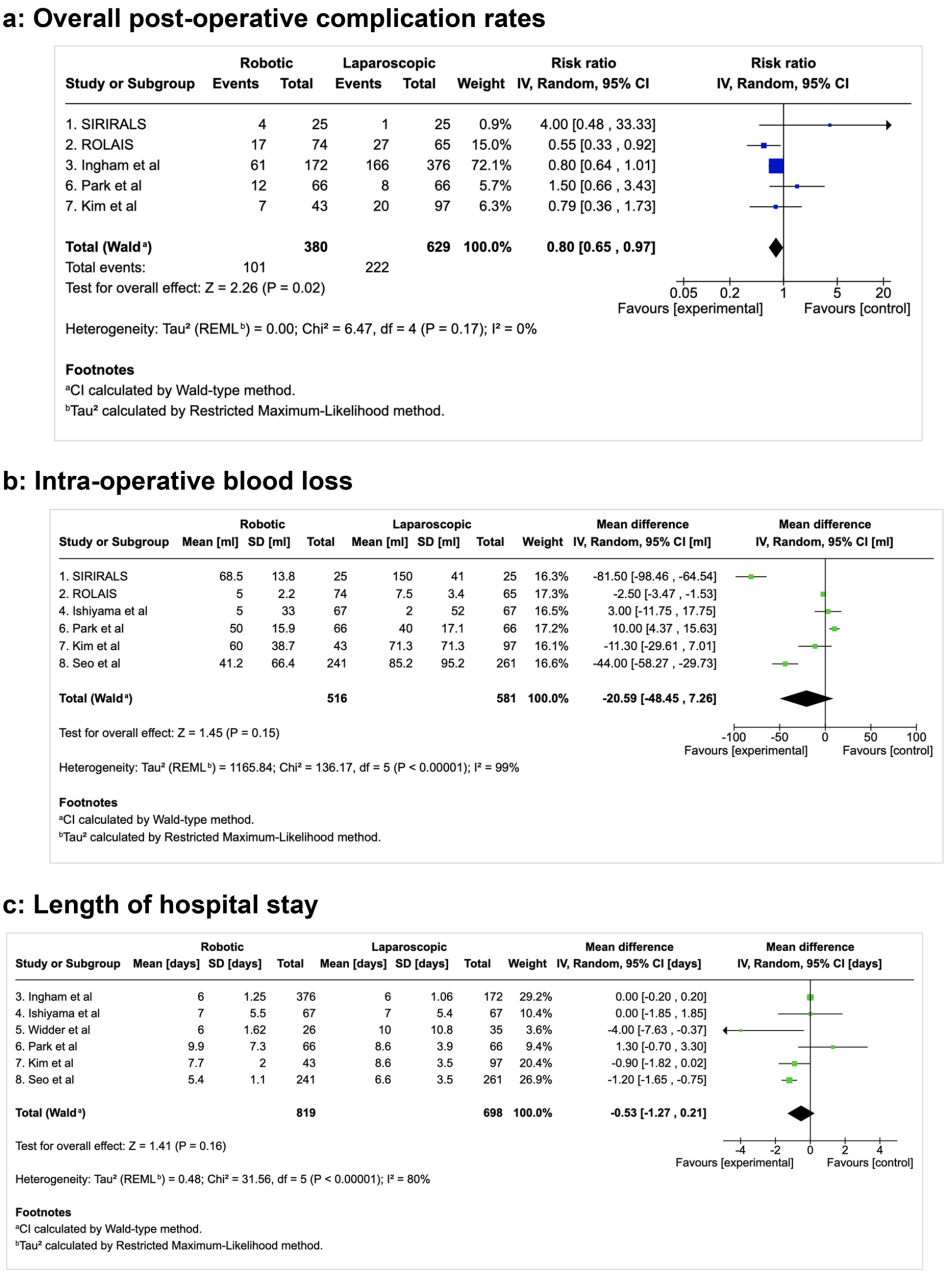
Comparison of clinical outcomes

The overall complication rate (*n* = 1009 patients; *n* = 380 robotic, *n* = 629 laparoscopic cases) was significantly lower in the robotic surgery group with no heterogeneity between studies (RR 0.8 95% CI [0.65, 0.97], *p* = 0.02; I² = 0%). Analysis of complication rates following colorectal cancer surgery specifically, (*n* = 870 patients; *n* = 306 robotic, *n* = 564 laparoscopic cases) [11, 12, 14, 15, 31] showed a non-significant trend towards a reduction in complication rates between the two approaches (RR 0.87, 95% CI [0.70, 1.08], *p* = 0.21; I² = 28%).

There was no significant difference in rates of major complications (CD > II) between the two approaches (RR 0.8, 95% CI [0.51, 1.26], *p* = 0.34 I² = 0%). It is important to mention that the majority of studies reported “major” complications as Clavien Dindo > II, that is to say significant complications that required surgical, endoscopic or radiological intervention. Of note, two studies classified “major” as Clavien Dindo > IIIb (re-intervention requiring general anaesthesia), thus not counting Clavien Dindo IIIa (re-intervention requiring local or regional anaesthesia) in their analysis [10, 15, 37]. Discounting these two studies, the findings remain consistent, as above, with no difference in the major complication rates following robotic versus laparoscopic surgery (RR 0.8, 95%CI [0.49, 1.51], *p* = 0.6; I² = 13%).

#### Secondary outcome measures: intra-operative blood loss (Fig. 4b)

Intra-operative blood loss data was available on 1,097 patients (*n* = 516 robotic, *n* = 581 laparoscopic cases). There was a non-significant trend towards reduced intra-operative blood loss in the robotic group, with significant heterogeneity noted (MD -20.59, 95% CI [-48.45, -7.26], *p* = 0.15 I² = 99%). The heterogeneity was significant (I² = 99%) and may be due to the procedural heterogeneity (both benign and cancer operations) included in the meta-analysis as well as differences in surgical technique and experience, patient and disease heterogeneity and variations in peri-operative care. Intra-operative blood loss did not differ between robotic and laparoscopic approach for colorectal cancer operations in subgroup analysis (*n* = 456 patients; *n* = 201 robotic, *n* = 255 laparoscopic cases) (MD 0.28, 95% CI [-4.57, 5.12], *p* = 0.91 I² = 97%).

#### Secondary outcome measures: length of stay (Fig. 4c)

Length of stay was reported on 1,517 patients (*n* = 819 robotic, *n* = 698 laparoscopic cases). Again, there was a non-significant trend towards a shorter length of stay following robotic surgery, with significant heterogeneity noted (MD -0.53, 95% CI [-1.27, 0.21], *p* = 0.16 I² = 80%). The heterogeneity was significant (I² = 80%) and may be due to the procedural heterogeneity (both benign and cancer operations) included in the meta-analysis, as well as differences in surgical technique and experience, patient and disease heterogeneity and variations in peri-operative care. Length of stay also did not differ significantly between robotic and laparoscopic approach for colorectal cancer operations in this subgroup analysis (*n* = 954 patients; *n* = 552 robotic, *n* = 402 laparoscopic cases) (MD -0.03, 95% CI [-0.22, 0.17], *p* = 0.78 I² = 43%).

## Discussion

This study identified that CRP serum levels measured on post-operative day three (POD3) were significantly lower following robotic compared to laparoscopic abdominal surgery. This finding was further demonstrated following robotic compared to laparoscopic colorectal cancer surgery.

This supports the hypothesis that the systemic inflammatory response that occurs as a result of surgical trauma may be lower following robotic surgery, potentially due to increased precision leading to reduced tissue stress and inflammation, in keeping with previous reports [17, 30, 38]. A reduced systemic inflammatory response may underpin the lower complication rate and more favourable clinical outcomes observed in robotic compared to laparoscopic surgery [30].

It is important to note that CRP alone is a non-specific acute phase reactant and marker of inflammation. CRP levels can be influenced not only by tissue trauma but also by complication rates, operative duration and complexity, patient comorbidities and clinical presentation, disease burden, conversion, perioperative care and institutional practice. Although this meta-analysis demonstrates that robotic surgery was associated with a statistically significant reduction in POD3 CRP (-13 mg/L overall *n* = 1,435 patients and − 17 mg/L in the colorectal cancer subgroup *n* = 872 patients), the clinical relevance of this difference should be interpreted cautiously. Regarding colorectal cancer patients, reported CRP thresholds associated with shorter disease free survival and worse oncological outcomes following surgery are typically substantially higher (associated with CRP levels approximately 125–150 mg/L on POD3) [39, 40]. However, there are smaller studies that report much smaller differences in CRP can have a statistically significant effect on cancer recurrence [5]. The observed effect in our study likely reflects a modest reduction in systemic inflammatory burden rather than a clinically decisive shift in postoperative risk classification. Whether this biochemical difference translates into meaningful improvements in postoperative outcomes remains uncertain.

The inflammatory response that occurs due to the trauma of a surgical procedure reflects global activation of the innate immune system [41]. This is characterised by a rise in serum WCC secondary to pro-inflammatory mediators promoting leucocyte migration, as well as cytokine release (especially IL-6) leading to a rise in CRP. There is also a drop in albumin secondary to increased vascular permeability, enhanced catabolism and inflammatory-related suppression of hepatic albumin synthesis [42, 43]. These biochemical surrogate markers of inflammation (high WCC, CRP and low albumin) are consistently associated with inflammatory burden and disease severity from both infectious and non-infectious causes of systemic inflammation [44–46].

The two randomised controlled trials included in this study demonstrated lower CRP post robotic surgery in keeping with a lower systemic inflammatory response [10, 31]. This was also reflected in four out of the six observational cohort studies demonstrating lower CRP or lower CRP/albumin ratios post robotic surgery [11–13, 15]. While one observational cohort study demonstrated equivocal post operative WCC, there was a better post-operative nutritional index as defined by [10 × serum albumin (g/dL)] + 0.005 × lymphocytes/µL] following robotic surgery [14]. The final observational cohort study demonstrated equivalence in post operative inflammation comparing conventional robotic versus conventional laparoscopic gastrectomy patients although secondary outcomes such as length of stay and return to soft diet were shorter following robotic surgery [16]. When combining the data from these studies in this meta-analysis, post-operative CRP levels were significantly lower on POD3 following robotic surgery in comparison with laparoscopy and maximum albumin levels were higher following robotic surgery thus supporting the hypothesis that robotic surgery may contribute to with a lower systemic inflammatory response [17, 30, 38].

A reduced post-operative inflammatory response, demonstrated by a lower post-operative CRP was also established within the robotic colorectal cancer subgroup in this meta-analysis. There is mounting evidence that a higher peri-operative inflammation is associated with poorer oncological outcomes in colorectal cancer [5, 6, 18, 47]. A high CRP, independent of post-operative complications, has been demonstrated to be associated with a shorter disease-free survival, shorter overall survival and higher risk of cancer recurrence [39, 40, 48]. While not directly assessed in this meta-analysis, other studies have shown that a higher peri-operative systemic inflammatory response, i.e. a more exaggerated pro-inflammatory state, may impair immunity potentially facilitating residual tumour cell survival, activation of circulating tumour cells and micro-metastatic progression [49–52].

Regarding secondary benefits of robotic surgery, this meta-analysis also demonstrated a trend toward reduced intraoperative blood loss with the robotic approach, consistent with published literature. This likely reflects reduced tissue trauma, enhanced visualization, and the improved dexterity afforded by the robotic platform [53–59]. Post-operative complication rates were significantly lower following robotic surgery in keeping with published literature [60–63]. While there was a trend in this meta-analysis towards reduced length of stay following robotic surgery, this did not reach significance in this meta-analysis, potentially due to the significant procedural heterogeneity included in the study. Published reports of reduced length of stay following robotic surgery [64, 65] may be attributable to lower rates of complications as well as reduced post-operative pain [12, 15, 27, 31, 66] and faster return of normal bowel function [15, 25, 67–69]. Operative duration using the robotic platform was significantly longer versus laparoscopy, which is consistent with existing published studies although this only reached borderline significance within the colorectal cancer subgroup [28, 54]. Despite the longer operative duration, the systemic inflammatory response remains lower in the robotic group. As technology, training pathways and surgical experiences advances; the robotic platform’s enhanced dexterity, visualisation, and ergonomics may mitigrate this difference in operative duration, particularly for complex cases such as advanced colorectal cancer resections.

### Strengths and limitations

The strengths of this meta-analysis are the deliberate focus on universally collected, objective measures, such as CRP, length of stay, and complication rates, to minimize the risk of detection bias and selective reporting bias. The authors also recognize that the overall analysis included both benign and malignant cases and given the research interest on the potential impact of perioperative inflammation on outcomes in colorectal cancer specifically, a dedicated subgroup analysis of colorectal cancer patients was undertaken to mitigate potential for reporting bias arising from this heterogeneity. In addition, no studies with a significant risk of bias were included in this meta-analysis (*please see supplementary material).*

A limitation of this study is that the inflammatory markers (CRP, WCC, albumin) are non-specific and can be influenced by multiple peri-operative factors such as infection, complication rates, pre-operative nutritional status and clinical presentation. CRP is an acute phase reactant and in itself is not a direct measure of surgical trauma, therefore as stated throughout this study; it is used as a surrogate marker for peri-operative inflammation.

A further limitation of this study is that not all studies initially identified provided CRP mean difference and standard deviation data, therefore corresponding authors were contacted to obtain additional information where possible. Nevertheless, CRP data was available for 1,435 patients which represents a substantial and methodologically robust sample size for evaluating the primary outcome. Given the retrospective nature of the observational studies, there is an inherent risk of selection bias within the cohort studies. Among the six cohort studies included, the authors generally did not specify whether outcome measurements and follow up was complete for all participants introducing the possibility of attrition bias.

## Conclusion

This systematic review and meta-analysis demonstrated post-operative CRP, as a biochemical surrogate marker of the systemic inflammatory response, was significantly lower following robotic surgery compared with laparoscopy. This finding supports the hypothesis that the enhanced precision, stability and refined tissue handling afforded by robotic surgical platforms may contribute to reduced tissue trauma and thus may contribute to a reduced post-operative inflammatory response. This result was consistent across all abdominal procedures included and within a colorectal cancer subgroup.

## Electronic Supplementary Material

Below is the link to the electronic supplementary material.

Supplement material 1

## Data Availability

No datasets were generated or analysed during the current study.
