# Supplementary material for: Systemic inflammatory response after robotic versus laparoscopic abdominal surgery: a systematic review and meta-analysis with colorectal cancer subgroup analysis
Source: J Robot Surg. 2026 Jun 1;20(1):565. doi: 10.1007/s11701-026-03527-x (PMC13226349; doi:10.1007/s11701-026-03527-x)
Supplement: Supplementary file 1 — Supplement material 1 [file 11701_2026_3527_MOESM1_ESM.docx]

## **Supplementary Figures**

- **Figure 1: Forest Plot: Primary Outcome Measure of CRP Day 1 Post-op**


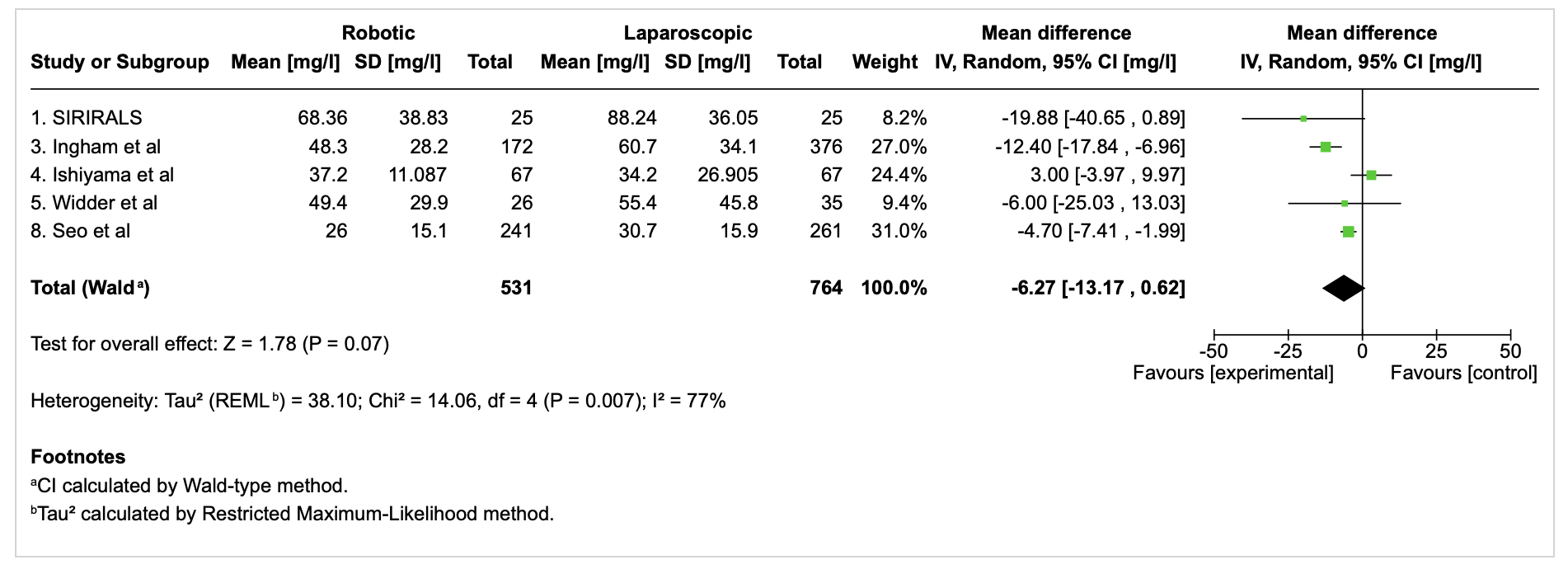


- **Figure 2: Forest Plot: Colorectal Cancer Subgroup: Primary Outcome Measure of CRP Day 1 Post-op**


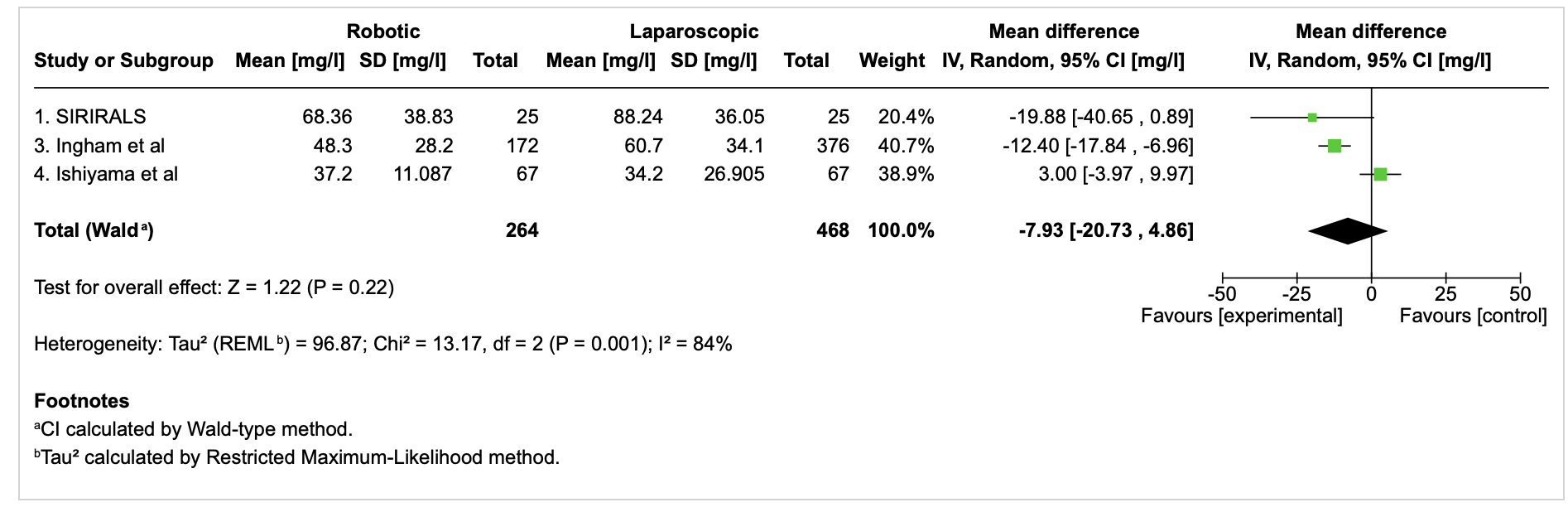


**Figure 3: Forest Plot: Primary Outcome Measure of Maximum WCC Post-op**


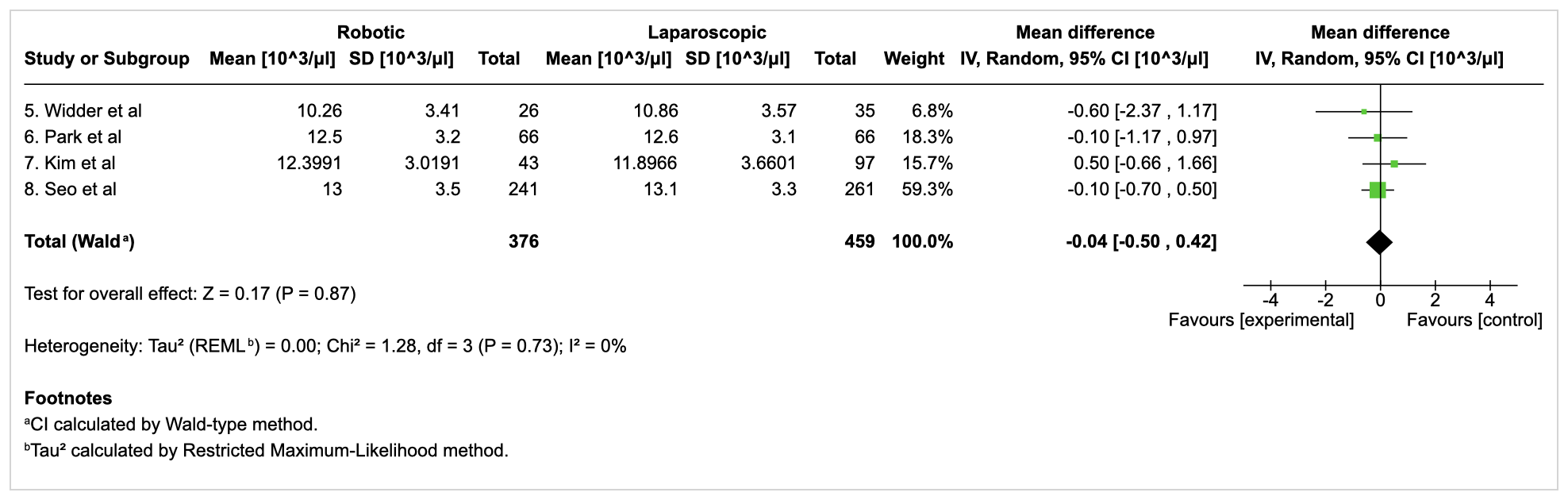


**Figure 4: Colorectal Cancer Subgroup: Secondary Outcome Measure of Major Complications**


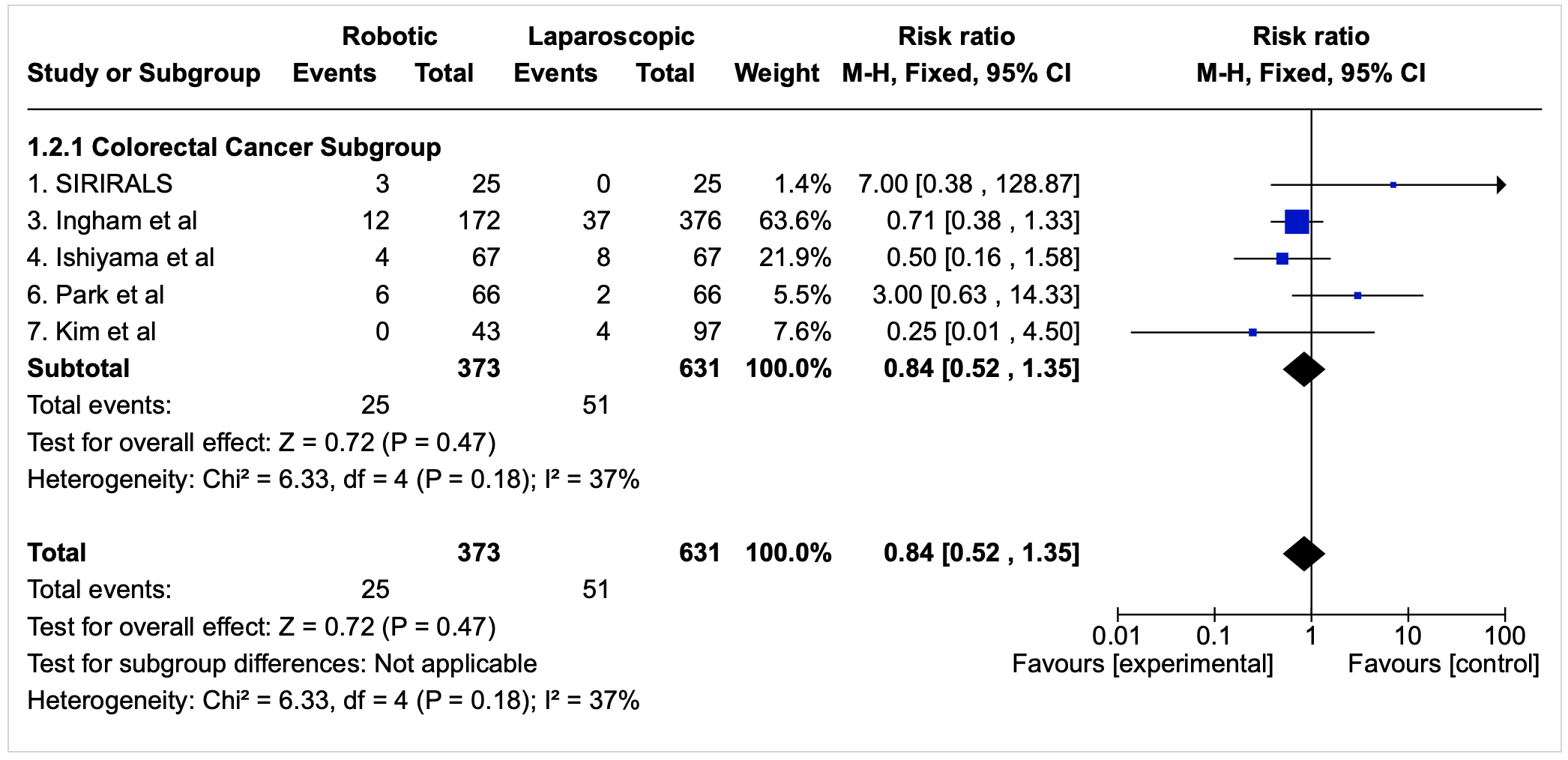


**Figure 5: Colorectal Cancer Subgroup: Secondary Outcome Measure of Length of Stay**

**
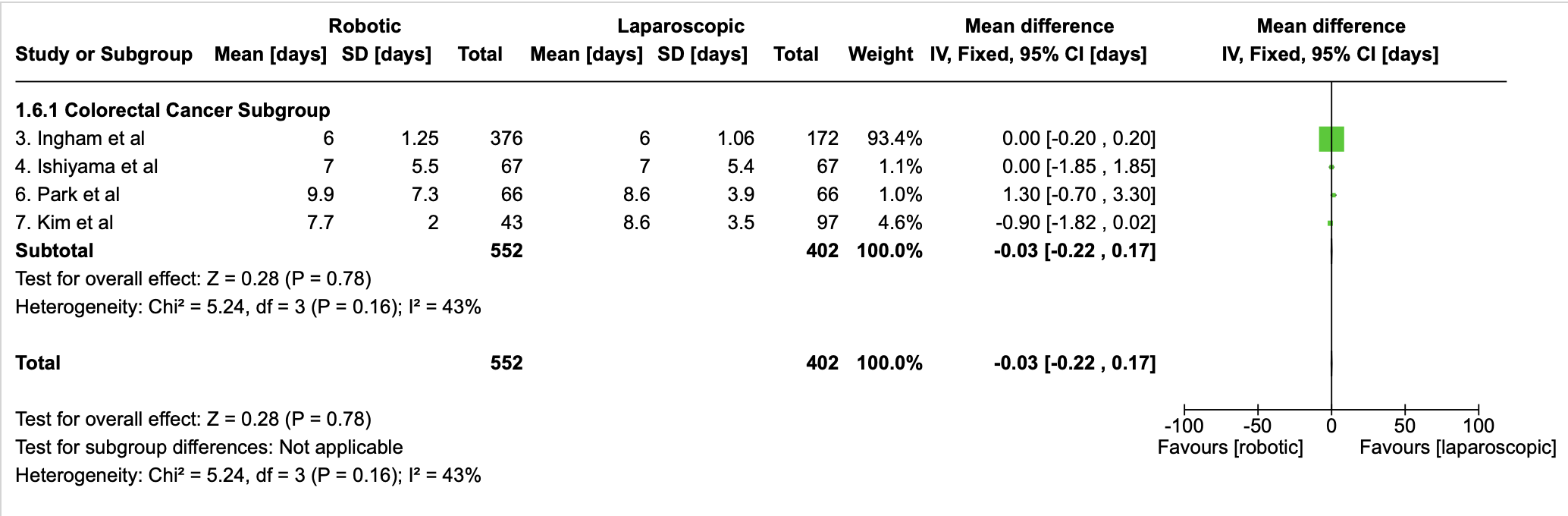
**

**Figure 6: Colorectal Cancer Subgroup: Secondary Outcome Measure of Intra-Operative Blood Loss**

**
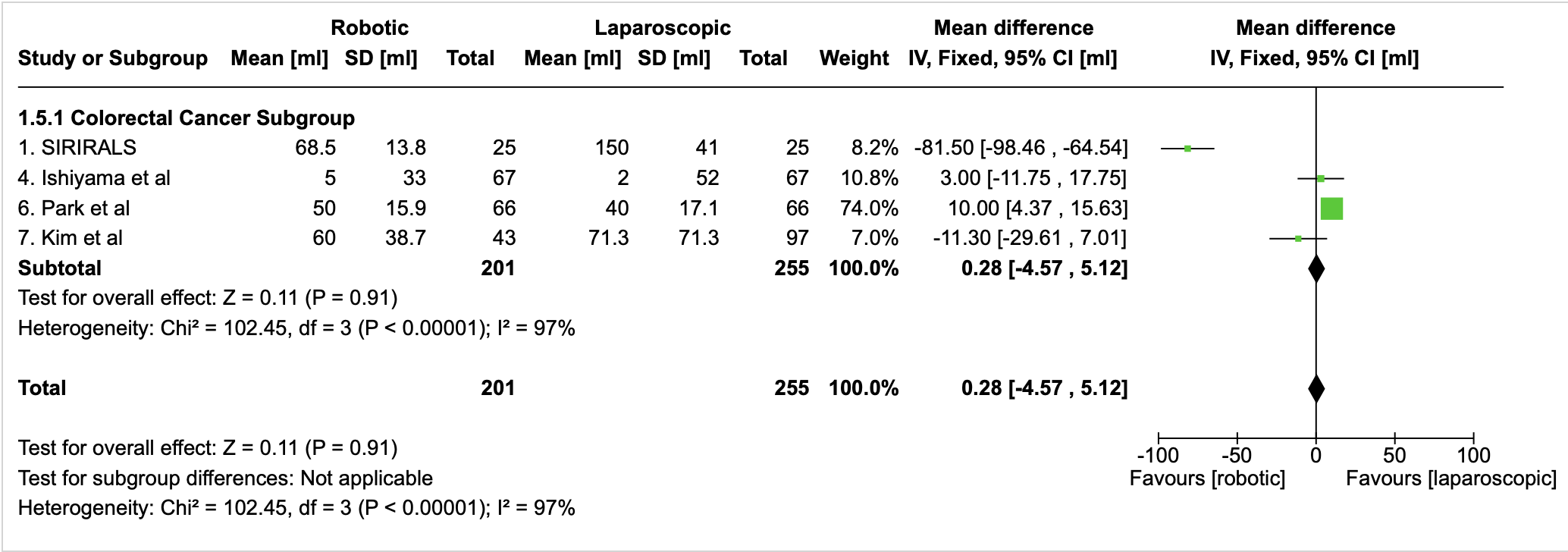
**

## **Risk-of-Bias Assessments**

All eight studies included in this systematic review and meta-analysis carried a moderate risk of bias *(see tables 1a-8c below with summary table 9a-d)*

Due to some concerns with risk of bias, the two randomised control trials (SIRIRALS & ROLAIS) carry a moderate strength of recommendation and quality of evidence. The six cohort studies have a moderate quality of evidence and strength of recommendation.

With regard to risk of bias due to missing results; only one of the cohort studies states that follow up was complete for all participants. Both randomised control trials state no participants were lost to follow up. For the remaining five cohort studies there is a risk of bias due to missing participant and outcome data in this regard. For the most part we did not find selective outcome reporting in the studies included in this systematic review, with the exception of the SIRIRALS trial did not which did not report on their pre-defined outcome measures of IL-6 post-operative results or length of stay. The majority of the studies included in this systematic review were of moderate to large sample size thus reducing bias and overestimation of treatment effects.

**Table 1a: Risk of Bias (RoB 2) Study 1: *Systemic inflammatory response in robot-assisted and laparoscopic colorectal surgery* (*SIRIRALS, Čuk P, et al)***

| **Domain** | **Risk of Bias** | **Explanation** |
| --- | --- | --- |
| Randomisation process | Some concerns | Only 50 patients were analysed out of 128 screened (potential for selection bias) & small sample size |
| Deviation from intended intervention | Low | Objective outcomes eg CRP & blinding of data assessors |
| Missing outcome data | Some concerns | Only 50 patients were analysed out of 128 screened, thus missing data could bias results |
| Measurement of outcome | Low | Primary outcome measures are objective biochemistry measurements & blinding of lab analysis  However some secondary outcome measures they report on such as pain scores are subjective therefore may be prone to expectation bias |
| Selection of the reported result | Some concerns | Primary outcome measure of IL-6 is not included in results – selective reporting. Neither is secondary outcome measure of length of stay |
| **Overall risk of bias** | **Some concerns** |  |

**Table 1b: RoB-ME Assessment: Study 1: *Systemic inflammatory response in robot-assisted and laparoscopic colorectal surgery* (*SIRIRALS, Čuk P, et al)***

| **Domain** | **Risk of Bias** | **Explanation** |
| --- | --- | --- |
| Missing outcome data | Some concerns | Only 50 patients were analysed out of 128 screened, thus missing data could bias results |
| Missing participant data | Low | Minimal withdrawals & no patients were lost to follow up |
| Selective outcome reporting | Some concerns | Primary outcome measure of IL-6 is not included in results – selective reporting. Neither is secondary outcome measure of length of stay |
| Small-study effects | Some concerns | Single centre trial, modest sample size could be sensitive to small study effects |
| Publication bias | Low | Registered trial with published results |
| **Overall** | **Some concerns for risk of bias due to missing results** |  |

**Table 1c: GRADE Assessment: Study 1 *Systemic inflammatory response in robot-assisted and laparoscopic colorectal surgery* (*SIRIRALS, Čuk P, et al)***

| **Domain** | **Assessment** | **Explanation** |
| --- | --- | --- |
| Risk of bias | Some concerns | *See table 1a* |
| Inconsistency | Some concerns | Primary outcome measure of IL-6 is not included in results – selective reporting. Neither is secondary outcome measure of length of stay |
| Indirectness | Low | The population, interventions, and outcomes are directly relevant to the clinical question |
| Imprecision | Some concerns | As only 50 participants, may have limited statistical power to detect small difference between the two groups. P-values and confidence intervals reported |
| Publication bias | Low | Registered trial with published results |
| **Overall confidence in quality of evidence** | **Moderate** |  |

Study 1: “Surgical stress response in robot-assisted versus laparoscopic surgery for colon cancer (SIRIRALS): randomized clinical trial” had some concerns regarding risk of bias as per RoB2 tool. Potential areas for bias within this study include selection bias; 75 participants out of 128 identified in screening were not included in the final analysis thus leading to a risk of missing outcome data. In addition IL-6 (one of the primary outcome measures) is not included in the analysis thus leaving potential for selective reporting. There were some concerns for risk of bias due to missing results as per RoB-ME Assessment tool, with an overall moderate confidence in quality of evidence as per GRADE Assessment tool.

**Table 2a: Risk of Bias (RoB 2) Study 2: *Inflammatory response and short-term outcomes after laparoscopic versus robotic transabdominal preperitoneal inguinal hernia repair: randomized clinical trial (ROLAIS, Valorenzos A, et al)***

| **Domain** | **Risk of Bias** | **Explanation** |
| --- | --- | --- |
| Randomisation process | Low | Computer generated randomisation using REDCap with similar group characteristics |
| Deviation from intended intervention | Some concerns | Open label thus room for performance bias in peri-operative care (eg decisions re overnight stay) |
| Missing outcome data | Some concerns | Difference in group size (74 R-TAPP versus 65 L-TAPP) could lead to differential attrition. Study does not mention whether there was any missing data of those analysed |
| Measurement of outcome | Low | Objective lab outcome measures (CRP) at defined time points. |
| Selection of the reported result | Low | Of note, registration of the trial occurred retrospectively May 2023 (trial began Nov 2022) |
| **Overall risk of bias** | **Some concerns** |  |

**Table 2b: RoB-ME Assessment: Study 2: *Inflammatory response and short-term outcomes after laparoscopic versus robotic transabdominal preperitoneal inguinal hernia repair: randomized clinical trial (ROLAIS).***

| **Domain** | **Risk of Bias** | **Explanation** |
| --- | --- | --- |
| Missing outcome data | Some concerns | Difference in group size (74 R-TAPP versus 65 L-TAPP) could lead to differential attrition with more drop-outs within L-TAPP group (n=9) after randomisation |
| Missing participant data | Low | No patients were lost to follow up |
| Selective outcome reporting | Low | All pre-specified primary and secondary outcome measures were reported |
| Small-study effects | Low | Moderate sample size for a surgical RCT |
| Publication bias | Some concerns | Trial registration occurred retrospectively May 2023 (trial began Nov 2022) |
| **Overall** | **Some concerns for risk of bias due to missing results** |  |

**Table 2c: GRADE Assessment: Study 2 *Inflammatory response and short-term outcomes after laparoscopic versus robotic transabdominal preperitoneal inguinal hernia repair: randomized clinical trial (ROLAIS).***

| **Domain** | **Assessment** | **Explanation** |
| --- | --- | --- |
| Risk of bias | Some concerns | *See table 2a* |
| Inconsistency | Low | Consistent findings reported for both groups & align with similar published studies |
| Indirectness | Low | The population, interventions, and outcomes are directly relevant to the clinical question |
| Imprecision | Low | Sufficient sample size, with p-values and confidence intervals reported. |
| Publication bias | Some concerns | Trial registration occurred retrospectively May 2023 (trial began Nov 2022) |
| **Overall confidence in quality of evidence** | **Moderate** |  |

Study 2: “Inflammatory response and short-term outcomes after laparoscopic versus robotic transabdominal preperitoneal inguinal hernia repair: randomized clinical trial (ROLAIS)” had some concerns regarding risk of bias as per RoB2 tool. The open-label nature of the study allows for some performance bias surrounding peri-operative decision making. In addition, the study does not include whether there was any missing data or if follow up was complete for all patients. The study began in November 2022, however was not registered on ClinicalTrials.gov until February 2023. There were some concerns for risk of bias due to missing results as per RoB-ME Assessment tool, with an overall moderate confidence in quality of evidence as per GRADE Assessment tool.

**Table 3a: Risk of Bias (ROBINS-1) Study 3: *Robotic-assisted surgery for left-sided colon and rectal resections is associated with reduction in the postoperative surgical stress response and improved short-term outcomes: a cohort study (Ingham AR, et al.)***

| **Domain** | **Risk of Bias** | **Explanation** |
| --- | --- | --- |
| Risk of bias due to confounding | Moderate | Single centre, surgeon experience allow for confounding bias. However there was comparable rates of complex cases between robotic versus laparoscopic cohort & case selection was adjusted for |
| Risk of bias in classification of interventions | Low | Intervention type is objective: robotic versus laparoscopic versus open surgery |
| Risk of bias in selection of participants into the study | Low | Patients are consecutive within time period |
| Risk of bias due to deviations from intended interventions | Moderate | Potential for difference in peri-operative management between groups, as is retrospective study there is no blinding. |
| Risk of bias due to missing data | Low | All patients had minimum follow up of 90 days |
| Risk of bias arising from measurement of the outcome | Low | Objective outcome measures CRP |
| Risk of bias in selection of the reported result | Low | While an observational study allows for selective reporting / choice of what outcome measures to include in analysis, this study included objective measures eg CRP and defined time points post-operatively |
| **Overall risk of bias** | **Moderate risk** |  |

**Table 3b: RoB-ME Assessment: Study 3: *Robotic-assisted surgery for left-sided colon and rectal resections is associated with reduction in the postoperative surgical stress response and improved short-term outcomes: a cohort study (Ingham AR, et al.)***

| **Domain** | **Risk of Bias** | **Explanation** |
| --- | --- | --- |
| Missing outcome data | Low | Large database, no indication that any outcome data was missing |
| Missing participant data | Low | All patients were followed up for minimum 90 days. Propensity score matching applied. |
| Selective outcome reporting | Low | This study reported all pre-specified outcome objective measures eg CRP at defined time points post-operatively |
| Small-study effects | Low | Large sample size |
| Publication bias | Unclear | Single cohort study, however is published in peer-reviewed journal |
| **Overall** | **Low risk of bias due to missing results** |  |

**Table 3c: GRADE Assessment: Study 3 *Robotic-assisted surgery for left-sided colon and rectal resections is associated with reduction in the postoperative surgical stress response and improved short-term outcomes: a cohort study (Ingham AR, et al.)***

| **Domain** | **Assessment** | **Explanation** |
| --- | --- | --- |
| Risk of bias | Moderate | *See table 3a* |
| Inconsistency | Low | Consistent findings reported for both groups & align with similar published studies |
| Indirectness | Low | The population, interventions, and outcomes are directly relevant to the clinical question |
| Imprecision | Some concerns | Large sample size, with p-values and confidence intervals reported. However some confidence intervals are wide indicating some imprecision |
| Publication bias | Unclear | Single observational cohort study |
| Magnitude of Effect | Moderate | Reports significant difference between the two groups, clinical significance requires further investigation |
| Plausible Confounding | Some concerns | Single centre, surgeon experience allow for confounding bias. However there was comparable rates of complex cases between robotic versus laparoscopic cohort & case selection was adjusted for |
| **Overall confidence in quality of evidence** | **Moderate** |  |

Study 3: “Robotic-assisted surgery for left-sided colon and rectal resections is associated with reduction in the postoperative surgical stress response and improved short-term outcomes: a cohort study” had a moderate risk of bias as per ROBINS-1 tool. It’s nature as a single centre observational study allows for some confounding bias with surgical experience impacting choice of approach. In addition, as it is a retrospective study, there may be selective reporting on what outcome measures to include and there is no mention of whether data collection and follow up was complete for all 1031 patients. These characteristics are true of all six retrospective cohort studies included in this systematic review. There were a low risk of bias due to missing results as per RoB-ME Assessment tool, with an overall moderate confidence in quality of evidence as per GRADE Assessment tool.

**Table 4a: Risk of Bias (ROBINS-1) Study 4: *Comparative analysis of postoperative inflammation and pain: robot-assisted versus single-incision laparoscopic surgery for right-sided colon cancer (Ishiyama Y, et al)***

| **Domain** | **Risk of Bias** | **Explanation** |
| --- | --- | --- |
| Risk of bias due to confounding | Moderate | Surgical approach was surgeon selected choice of approach & seems based on availability of robotic console leading to case selection bias |
| Risk of bias in classification of interventions | Low | Intervention type is objective: robotic versus single incision laparoscopy |
| Risk of bias in selection of participants into the study | Low | Patients are consecutive within time period |
| Risk of bias due to deviations from intended interventions | Moderate | Potential for difference in peri-operative management between groups, as is retrospective study there is no blinding & could impact on subjective reporting of pain scores |
| Risk of bias due to missing data | Moderate | There is no mention of if data collection was complete or follow up complete |
| Risk of bias arising from measurement of the outcome | Moderate | Inflammation markers eg CRP are objective, however pain scores are subjective therefore prone to expectation bias |
| Risk of bias in selection of the reported result | Low | While an observational study allows for selective reporting / choice of what outcome measures to include in analysis, this study included objective measures eg CRP and defined time points post-operatively |
| **Overall risk of bias** | **Moderate risk** |  |

**Table 4b: RoB-ME Assessment: Study 4: *Comparative analysis of postoperative inflammation and pain: robot-assisted versus single-incision laparoscopic surgery for right-sided colon cancer (Ishiyama Y, et al)***

| **Domain** | **Risk of Bias** | **Explanation** |
| --- | --- | --- |
| Missing outcome data | Low | No indication that outcome data is missing |
| Missing participant data | Some concerns | There is no mention of if follow up complete. Of note, propensity matching was applied. |
| Selective outcome reporting | Low | This study reported all pre-specified outcome objective measures eg CRP at defined time points post-operatively |
| Small-study effects | Low | Moderate sample size for cohort study |
| Publication bias | Unclear | Single cohort study, however is published in peer-reviewed journal |
| **Overall** | **Some concerns for risk of bias due to missing results** |  |

**Table 4c: GRADE Assessment: Study 4 *Comparative analysis of postoperative inflammation and pain: robot-assisted versus single-incision laparoscopic surgery for right-sided colon cancer (Ishiyama Y, et al)***

| **Domain** | **Assessment** | **Explanation** |
| --- | --- | --- |
| Risk of bias | Moderate risk of bias | *See table 4a* |
| Inconsistency | Low | Consistent findings reported for both groups & align with similar published studies |
| Indirectness | Low | The population, interventions, and outcomes are directly relevant to the clinical question |
| Imprecision | Some concerns | Sufficient sample size, with p-values and confidence intervals reported. However some confidence intervals are wide indicating some imprecision |
| Publication bias | Unclear | Single observational cohort study |
| Magnitude of Effect | Moderate | Reports significant difference between the two groups suggesting clinically meaningful effect |
| Plausible Confounding | Moderate | Surgical approach was surgeon selected choice of approach & seems based on availability of robotic console leading to case selection bias |
| **Overall confidence in quality of evidence** | **Moderate** |  |

Study 4: “Comparative analysis of postoperative inflammation and pain: robot-assisted versus single-incision laparoscopic surgery for right-sided colon cancer” had a moderate risk of bias as per ROBINS-1 tool. This was predominantly due to surgeon selection of operative approach, based on availability of robotic console as well as one of the primary outcome measures (numerical pain scores) being subjective. There were some concerns for risk of bias due to missing results as per RoB-ME Assessment tool, with an overall moderate confidence in quality of evidence as per GRADE Assessment tool.

**Table 5a: Risk of Bias (ROBINS-1) Study 5: *Robotic-Assisted versus Laparoscopic Left Hemicolectomy-Postoperative Inflammation Status, Short-Term Outcome and Cost Effectiveness (Widder A, et al)***

| **Domain** | **Risk of Bias** | **Explanation** |
| --- | --- | --- |
| Risk of bias due to confounding | Moderate | Surgical approach was divided into those operated on (laparoscopically) before and after (robotic) the introduction of the robotic platform halfway through the study period. The learning curve is not accounted for & two surgeons operate using the both platforms, while one surgeon performs laparoscopic approach only. |
| Risk of bias in classification of interventions | Low | Intervention type is objective: robotic versus laparoscopic |
| Risk of bias in selection of participants into the study | Moderate | While it is a consecutive series, selection into each group is not controlled as mentioned above. |
| Risk of bias due to deviations from intended interventions | Moderate | Potential for difference in peri-operative management between groups, as is retrospective study there is no blinding. In addition, peri-operative management may have changed over the two different time periods |
| Risk of bias due to missing data | Moderate | There is no mention of if data collection was complete or follow up complete |
| Risk of bias arising from measurement of the outcome | Low | Objective outcome measures including length of stay, cost analysis and biochemical data. |
| Risk of bias in selection of the reported result | Moderate | While an observational study allows for selective reporting / choice of what outcome measures to include in analysis, this study included objective measures eg length of stay and biochemical data. There may be some subjectivity on reporting of financial data. |
| **Overall risk of bias** | **Moderate risk** |  |

**Table 5b: RoB-ME Assessment: Study 5: *Robotic-Assisted versus Laparoscopic Left Hemicolectomy-Postoperative Inflammation Status, Short-Term Outcome and Cost Effectiveness (Widder A, et al)***

| **Domain** | **Risk of Bias** | **Explanation** |
| --- | --- | --- |
| Missing outcome data | Low | No indication that outcome data is missing |
| Missing participant data | Some concerns | There is no mention of if follow up complete. Of note, propensity matching was applied. |
| Selective outcome reporting | Low | This study reported all pre-specified outcome objective measures eg length of stay, biochemical data |
| Small-study effects | Some concerns | Small sample size could allow for overestimation of treatment effects |
| Publication bias | Unclear | Single cohort study, however is published in peer-reviewed journal |
| **Overall** | **Some concerns for risk of bias due to missing results** |  |

**Table 5c: GRADE Assessment: Study 5 *Robotic-Assisted versus Laparoscopic Left Hemicolectomy-Postoperative Inflammation Status, Short-Term Outcome and Cost Effectiveness (Widder A, et al)***

| **Domain** | **Assessment** | **Explanation** |
| --- | --- | --- |
| Risk of bias | Moderate | *See table 5a* |
| Inconsistency | Low | Consistent findings reported for both groups & align with similar published studies |
| Indirectness | Low | The population, interventions, and outcomes are directly relevant to the clinical question |
| Imprecision | Some concerns | Small sample size which may limit precision of results and confidence intervals are not provided for primary outcome measures |
| Publication bias | Unclear | Single observational cohort study |
| Magnitude of Effect | Moderate | Reports significant difference between the two groups suggesting clinically meaningful effect |
| Plausible Confounding | Moderate | Surgical approach was divided into those operated on (laparoscopically) before and after (robotic) the introduction of the robotic platform halfway through the study period. The learning curve is not accounted for & two surgeons operate using the both platforms, while one surgeon performs laparoscopic approach only. |
| **Overall confidence in quality of evidence** | **Moderate** |  |

Study 5: “Robotic-Assisted versus Laparoscopic Left Hemicolectomy-Postoperative Inflammation Status, Short-Term Outcome and Cost Effectiveness.” had a moderate risk of bias as per ROBINS-1 tool. The study period included consecutive patients, however the groups were divided into those operated on via a laparoscopic approach in the first half of the study time period, and following the introduction of the robotic platform, the latter half of the study group were operated on using a robotic approach. This leads to uncontrolled confounding bias*.* There were some concerns for risk of bias due to missing results as per RoB-ME Assessment tool, with an overall moderate confidence in quality of evidence as per GRADE Assessment tool.

**Table 6a: Risk of Bias (ROBINS-1) Study 6: *Robotic surgery may lead to reduced postoperative inflammatory stress in colon cancer: a propensity score–matched analysis (Park EJ, et al.)***

| **Domain** | **Risk of Bias** | **Explanation** |
| --- | --- | --- |
| Risk of bias due to confounding | Moderate | Propensity score matching was applied. However surgeon experience may lead to confounding bias |
| Risk of bias in classification of interventions | Low | Intervention type is objective: robotic versus laparoscopic |
| Risk of bias in selection of participants into the study | Moderate | Propensity score matching was applied. Surgical preference for approach may lead to selection bias |
| Risk of bias due to deviations from intended interventions | Moderate | Operations that were converted to open were analysed as initially intended approach. Potential for difference in peri-operative management between groups (eg resumption of soft diet) as is retrospective study there is no blinding. |
| Risk of bias due to missing data | Moderate | While median follow up was similar for both groups, it does not mention if any patients were lost to follow up |
| Risk of bias arising from measurement of the outcome | Moderate | Objective outcome measures including biochemical data, length of stay. However some subjective reporting data eg time to first flatus and resumption of diet – potential for cognitive bias |
| Risk of bias in selection of the reported result | Low | While an observational study allows for selective reporting / choice of what outcome measures to include in analysis, this study included objective measures eg CRP and length of stay. |
| **Overall risk of bias** | **Moderate risk** |  |

**Table 6b: RoB-ME Assessment: Study 6: *Robotic surgery may lead to reduced postoperative inflammatory stress in colon cancer: a propensity score–matched analysis (Park EJ, et al.)***

| **Domain** | **Risk of Bias** | **Explanation** |
| --- | --- | --- |
| Missing outcome data | Low | No indication that outcome data is missing |
| Missing participant data | Some concerns | Propensity matching was applied. While median follow-up for both groups was similar, it does not mention if any patients were lost to follow up |
| Selective outcome reporting | Low | This study reported all pre-specified outcome objective measures eg biochemical data, length of stay, time to first flatus and resumption of diet |
| Small-study effects | Low | Moderate sample size for cohort study |
| Publication bias | Unclear | Single cohort study, however is published in peer-reviewed journal |
| **Overall** | **Some concerns for risk of bias due to missing results** |  |

**Table 6c: GRADE Assessment: Study 6 *Robotic surgery may lead to reduced postoperative inflammatory stress in colon cancer: a propensity score–matched analysis (Park EJ, et al.)***

| **Domain** | **Assessment** | **Explanation** |
| --- | --- | --- |
| Risk of bias | Moderate | *See table 6a* |
| Inconsistency | Low | Consistent findings reported for both groups & align with similar published studies |
| Indirectness | Low | The population, interventions, and outcomes are directly relevant to the clinical question |
| Imprecision | Some concerns | Moderate sample size for cohort study, confidence intervals not provided |
| Publication bias | Unclear | Single observational cohort study |
| Magnitude of Effect | Moderate | Reports minimal difference between the two groups, however the effect sizes are clinically meaningful |
| Plausible Confounding | Moderate | Propensity score matching was applied. However surgeon experience may lead to confounding bias |
| **Overall confidence in quality of evidence** | **Moderate** |  |

Study 6: “Robotic surgery may lead to reduced postoperative inflammatory stress in colon cancer: a propensity score–matched analysis” had a moderate risk of bias as per ROBINS-1 tool. While propensity score matching was applied balancing observed confounders, surgeon experience and preference may lead to selection bias and there is room for potential reporting errors with outcome measures (eg patient reporting of time to first flatus). There were some concerns for risk of bias due to missing results as per RoB-ME Assessment tool, with an overall moderate confidence in quality of evidence as per GRADE Assessment tool.

**Table 7a: Risk of Bias (ROBINS-1) Study 7: *Short‑term outcomes of single‑incision robotic colectomy versus conventional multiport laparoscopic colectomy for colon cancer (Kim et al)***

| **Domain** | **Risk of Bias** | **Explanation** |
| --- | --- | --- |
| Risk of bias due to confounding | Moderate | Propensity score matching was applied, residual confounding may exist due to unmeasured variables |
| Risk of bias in classification of interventions | Low | Intervention type is objective: robotic versus laparoscopic |
| Risk of bias in selection of participants into the study | Moderate | Propensity score matching was applied. However operative approach was patient preference driven |
| Risk of bias due to deviations from intended interventions | Moderate | Potential for difference in peri-operative management between groups, as is retrospective study there is no blinding |
| Risk of bias due to missing data | Moderate | There is no mention of if data collection was complete for all patients |
| Risk of bias arising from measurement of the outcome | Low | Objective outcome measures including complication rates & biochemical data |
| Risk of bias in selection of the reported result | Low | There did not appear to be any selective reporting |
| **Overall risk of bias** | **Moderate risk** |  |

**Table 7b: RoB-ME Assessment: Study 7: *Short‑term outcomes of single‑incision robotic colectomy versus conventional multiport laparoscopic colectomy for colon cancer (Kim et al)***

| **Domain** | **Risk of Bias** | **Explanation** |
| --- | --- | --- |
| Missing outcome data | Low | No indication that outcome data is missing |
| Missing participant data | Some concerns | There is no mention of if follow up complete. Of note, propensity matching was applied. |
| Selective outcome reporting | Low | This study reported all pre-specified outcome objective measures eg biochemical data, complication rates |
| Small-study effects | Low | Moderate sample size for cohort study |
| Publication bias | Unclear | Single cohort study, however is published in peer-reviewed journal |
| **Overall** | **Some concerns for risk of bias due to missing results** |  |

**Table 7c: GRADE Assessment: Study 7 *Short‑term outcomes of single‑incision robotic colectomy versus conventional multiport laparoscopic colectomy for colon cancer (Kim et al)***

| **Domain** | **Assessment** | **Explanation** |
| --- | --- | --- |
| Risk of bias | Moderate | *See table 7a* |
| Inconsistency | Low | Consistent findings reported for both groups & align with similar published studies |
| Indirectness | Low | The population, interventions, and outcomes are directly relevant to the clinical question |
| Imprecision | Some concerns | Moderate sample size for cohort study, confidence intervals not provided |
| Publication bias | Unclear | Single observational cohort study |
| Magnitude of Effect | Moderate | Reports significant difference between the two groups suggesting clinically meaningful effect |
| Plausible Confounding | Moderate | Propensity score matching was applied, residual confounding may exist due to unmeasured variables |
| **Overall confidence in quality of evidence** | **Moderate** |  |

Study 7: “Short‑term outcomes of single‑incision robotic colectomy versus conventional multiport laparoscopic colectomy for colon cancer” had a moderate risk of bias as per ROBINS-1 tool. While propensity score matching was applied balancing observed confounders, operative approach was decided based on patient preference. There were some concerns for risk of bias due to missing results as per RoB-ME Assessment tool, with an overall moderate confidence in quality of evidence as per GRADE Assessment tool.

**Table 8a: Risk of Bias (ROBINS-1) Study 8: *Reduced‑port totally robotic distal subtotal gastrectomy for gastric cancer: 100 consecutive cases in comparison with conventional robotic and laparoscopic distal subtotal gastrectomy (Seo et al)***

| **Domain** | **Risk of Bias** | **Explanation** |
| --- | --- | --- |
| Risk of bias due to confounding | Moderate | Propensity score matching was applied, residual confounding may exist due to unmeasured variables |
| Risk of bias in classification of interventions | Low | Intervention type is objective: robotic versus laparoscopic |
| Risk of bias in selection of participants into the study | Moderate | The study does not report on patient selection for each operative approach - unclear |
| Risk of bias due to deviations from intended interventions | Moderate | Potential for difference in peri-operative management between groups, as is retrospective study there is no blinding |
| Risk of bias due to missing data | Moderate | There is no mention of if data collection was complete for all patients |
| Risk of bias arising from measurement of the outcome | Low | Objective outcome measures including biochemical data, complications rates, length of stay. |
| Risk of bias in selection of the reported result | Low | There did not appear to be any selective reporting |
| **Overall risk of bias** | **Moderate risk** |  |

**Table 8b: RoB-ME Assessment: Study 8: *Reduced‑port totally robotic distal subtotal gastrectomy for gastric cancer: 100 consecutive cases in comparison with conventional robotic and laparoscopic distal subtotal gastrectomy (Seo et al)***

| **Domain** | **Risk of Bias** | **Explanation** |
| --- | --- | --- |
| Missing outcome data | Low | No indication that outcome data is missing |
| Missing participant data | Some concerns | There is no mention of if follow up complete. Of note, propensity matching was applied. |
| Selective outcome reporting | Low | This study reported all pre-specified outcome objective measures eg biochemical data, complication rates, length of stay |
| Small-study effects | Low | Large sample size for cohort study |
| Publication bias | Unclear | Single cohort study, however is published in peer-reviewed journal |
| **Overall** | **Some concerns for risk of bias due to missing results** |  |

**Table 8c: GRADE Assessment: Study 8 *Reduced‑port totally robotic distal subtotal gastrectomy for gastric cancer: 100 consecutive cases in comparison with conventional robotic and laparoscopic distal subtotal gastrectomy (Seo et al)***

| **Domain** | **Assessment** | **Explanation** |
| --- | --- | --- |
| Risk of bias | Moderate | *See table 8a* |
| Inconsistency | Low | Consistent findings reported for both groups & align with similar published studies |
| Indirectness | Low | The population, interventions, and outcomes are directly relevant to the clinical question |
| Imprecision | Low | Large sample size, with p-values and standard deviations reported. |
| Publication bias | Unclear | Single observational cohort study |
| Magnitude of Effect | Moderate | Reports significant difference between the two groups suggesting clinically meaningful effect |
| Plausible Confounding | Moderate | Propensity score matching was applied, residual confounding may exist due to unmeasured variables |
| **Overall confidence in quality of evidence** | **Moderate** |  |

Study 8: “Reduced‑port totally robotic distal subtotal gastrectomy for gastric cancer: 100 consecutive cases in comparison with conventional robotic and laparoscopic distal subtotal gastrectomy” had a moderate risk of bias as per ROBINS-1 tool. There was no data surrounding patient selection into each group included in the paper ad while propensity score matching was applied, there may be residual confounding due to unmeasured variables. There were some concerns for risk of bias due to missing results as per RoB-ME Assessment tool, with an overall moderate confidence in quality of evidence as per GRADE Assessment tool.

**Table 9a: Summary of Risk of Bias Assessments for Randomised Control Trials (RoB 2)**

| **STUDY NUMBER & NAME** | **1**  **SIRIRALS** | **2**  **ROLAIS** |
| --- | --- | --- |
| **Domain** | | |
| Randomisation process | 🟡 | 🟢 |
| Deviation from intended intervention | 🟢 | 🟡 |
| Missing outcome data | 🟡 | 🟡 |
| Measurement of outcome | 🟢 | 🟢 |
| Selection of the reported result | 🟡 | 🟢 |
| **Overall risk of bias** | 🟡 | 🟡 |

🟢 Low risk 🟡 Some concerns 🔴 High risk

**Table 9b: Summary of Risk of Bias Assessments for Cohort Studies (ROBINS-1)**

| **STUDY NUMBER & NAME** | **3**  **Ingham et al** | **4**  **Ishiyama et al** | **5**  **Widder et al** | **6**  **Park et al** | **7**  **Kim et al** | **8**  **Seo et al** |
| --- | --- | --- | --- | --- | --- | --- |
| Risk of bias due to confounding | 🟡 | 🟡 | 🟡 | 🟡 | 🟡 | 🟡 |
| Risk of bias in classification of interventions | 🟢 | 🟢 | 🟢 | 🟢 | 🟢 | 🟢 |
| Risk of bias in selection of participants into the study | 🟢 | 🟢 | 🟡 | 🟡 | 🟡 | 🟡 |
| Risk of bias due to deviations from intended interventions | 🟡 | 🟡 | 🟡 | 🟡 | 🟡 | 🟡 |
| Risk of bias due to missing data | 🟢 | 🟡 | 🟡 | 🟡 | 🟡 | 🟡 |
| Risk of bias arising from measurement of the outcome | 🟢 | 🟡 | 🟢 | 🟡 | 🟢 | 🟢 |
| Risk of bias in selection of the reported result | 🟢 | 🟢 | 🟡 | 🟢 | 🟢 | 🟢 |
| **Overall risk of bias** | 🟡 | 🟡 | 🟡 | 🟡 | 🟡 | 🟡 |

🟢 Low risk 🟡 Moderate risk 🔴 Serious risk

**Table 9c: Summary of Risk of Bias from Missing Evidence (RoB-ME) Assessment**

| **STUDY NUMBER & NAME** | **1**  **SIRIRALS** | **2**  **ROLAIS** | **3**  **Ingham et al** | **4**  **Ishiyama et al** | **5**  **Widder et al** | **6**  **Park et al** | **7**  **Kim et al** | **8**  **Seo et al** |
| --- | --- | --- | --- | --- | --- | --- | --- | --- |
| Missing outcome data | 🟡 | 🟡 | 🟢 | 🟢 | 🟢 | 🟢 | 🟢 | 🟢 |
| Missing participant data | 🟢 | 🟢 | 🟢 | 🟡 | 🟡 | 🟡 | 🟡 | 🟡 |
| Selective outcome reporting | 🟡 | 🟢 | 🟢 | 🟢 | 🟢 | 🟢 | 🟢 | 🟢 |
| Small-study effects | 🟡 | 🟢 | 🟢 | 🟢 | 🟡 | 🟢 | 🟢 | 🟢 |
| Publication bias | 🟢 | 🟡 | - | - | - | - | - | - |
| **Overall risk of bias** | 🟡 | 🟡 | 🟢 | 🟡 | 🟡 | 🟡 | 🟡 | 🟡 |

🟢 Low risk 🟡 Some concerns 🔴 High risk

**Table 9d: Summary of GRADE Assessment for quality of evidence and strength of recommendations**

| **STUDY NUMBER & NAME** | **1**  **SIRIRALS** | **2**  **ROLAIS** | **3**  **Ingham et al** | **4**  **Ishiyama et al** | **5**  **Widder et al** | **6**  **Park et al** | **7**  **Kim et al** | **8**  **Seo et al** |
| --- | --- | --- | --- | --- | --- | --- | --- | --- |
| Risk of bias | 🟡 | 🟡 | 🟡 | 🟡 | 🟡 | 🟡 | 🟡 | 🟡 |
| Inconsistency | 🟡 | 🟢 | 🟢 | 🟢 | 🟢 | 🟢 | 🟢 | 🟢 |
| Indirectness | 🟢 | 🟢 | 🟢 | 🟢 | 🟢 | 🟢 | 🟢 | 🟢 |
| Imprecision | 🟡 | 🟢 | 🟡 | 🟡 | 🟡 | 🟡 | 🟡 | 🟢 |
| Publication bias | 🟢 | 🟡 | - | - | - | - | - | - |
| Magnitude of Effect | - | - | 🟡 | 🟡 | 🟡 | 🟡 | 🟡 | 🟡 |
| Plausible Confounding | - | - | 🟡 | 🟡 | 🟡 | 🟡 | 🟡 | 🟡 |
| **Overall confidence in quality of evidence** | 🟡 | 🟡 | 🟡 | 🟡 | 🟡 | 🟡 | 🟡 | 🟡 |

🟢 High confidence 🟡 Moderate confidence 🔴 Low confidence
